# Ethanolic Extract of *Moringa oleifera* Leaves Influences NF-κB Signaling Pathway to Restore Kidney Tissue from Cobalt-Mediated Oxidative Injury and Inflammation in Rats

**DOI:** 10.3390/nu12041031

**Published:** 2020-04-09

**Authors:** Mohamed M. Abdel-Daim, Samah R. Khalil, Ashraf Awad, Ehsan H. Abu Zeid, Reda Abd El-Aziz, Hamed A. El-Serehy

**Affiliations:** 1 Department of Zoology, College of Science, King Saud University, P.O. Box 2455, Riyadh 11451, Saudi Arabia; abdeldaim.m@vet.suez.edu.eg (M.M.A.-D.); helserehy@ksu.edu.sa (H.A.E.-S.); 2 Pharmacology Department, Faculty of Veterinary Medicine, Suez Canal University, Ismailia 41522, Egypt; 3 Forensic Medicine and Toxicology Department, Faculty of Veterinary Medicine, Zagazig University, Zagazig 44511, Egypt; ehelsharkawy@zu.edu.eg; 4 Animal wealth Development Department, Faculty of Veterinary Medicine, Zagazig University, Zagazig 44511, Egypt; afsawad@zu.edu.eg; 5 Physiology Department, Faculty of Veterinary Medicine, Zagazig University, Zagazig 44511, Egypt; mmreda@zu.edu.eg

**Keywords:** *Moringa oleifera*, cobalt, NF-κB, myeloperoxidase, C-reactive protein, TNF-α, IL-6, 8-hydroxy-2-deoxyguanosine

## Abstract

This study aimed to describe the protective efficacy of *Moringa oleifera* ethanolic extract (MOEE) against the impact of cobalt chloride (CoCl_2_) exposure on the rat’s kidney. Fifty male rats were assigned to five equal groups: a control group, a MOEE-administered group (400 mg/kg body weight (bw), daily via gastric tube), a CoCl_2_-intoxicated group (300 mg/L, daily in drinking water), a protective group, and a therapeutic co-administered group that received MOEE prior to or following and concurrently with CoCl_2_, respectively. The antioxidant status indices (superoxide dismutase (SOD), catalase (CAT), and reduced glutathione (GSH)), oxidative stress markers (hydrogen peroxide (H_2_O_2_), 8-hydroxy-2-deoxyguanosine (8-OHdG), and malondialdehyde (MDA)), and inflammatory response markers (nitric oxide (NO), tumor necrosis factor (TNF-α), myeloperoxidase (MPO), and C-reactive protein (CRP)) were evaluated. The expression profiles of pro-inflammatory cytokines (nuclear factor-kappa B (NF-kB) and interleukin-6 (IL-6)) were also measured by real-time quantitative polymerase chain reaction (qRT-PCR). The results showed that CoCl_2_ exposure was associated with significant elevations of oxidative stress and inflammatory indices with reductions in the endogenous tissue antioxidants’ concentrations. Moreover, CoCl_2_ enhanced the activity of the NF-κB inflammatory-signaling pathway that plays a role in the associated inflammation of the kidney. MOEE ameliorated CoCl_2_-induced renal oxidative damage and inflammatory injury with the suppression of the mRNA expression pattern of pro-inflammatory cytokine-encoding genes. MOEE is more effective when it is administered with CoCl_2_ exposure as a prophylactic regimen. In conclusion, MOEE administration exhibited protective effects in counteracting CoCl_2_-induced renal injury in rats.

## 1. Introduction

Cobalt (Co^2+^) is a trace element that is involved in the normal physiological functions of the nervous system and bone marrow erythropoiesis. Therefore, Co^2+^ nutritional deficiency may manifest as pernicious anemia, growth retardation, digestive disturbances, and myelination defects [1]. Yet, excessive exposure of humans and animals to high concentrations of Co^2+^ can occur through the inhalation of contaminated air and dietary routes as consumption of contaminated food, water, and cobalt-containing supplements. Numerous cases of Co^2+^ toxicity have been reported following occupational exposure and the use of industrial products as electrochemical products, rechargeable batteries, ceramics, cemented tungsten–cobalt, paints, and varnishes [2]. Over-the-counter supplements and illegal doping of athletes and racehorses with cobalt-containing products are potential sources of excessive exposure [3]. Further, cobalt has been used in various metal dental alloys, such as porcelain-fused-to-metal crowns, bridges, and dental implants [4]. The release of particulate wear debris from Co^2+^ alloys into the blood and various tissues, including the liver and kidneys, results in local cell toxicity and tissue infiltration with macrophages, which lead to bone and soft tissue injuries, as well as sensory and motor impairments [5]. Pan et al. found that cobalt–chrome alloys cause transient trace metal accumulation and apoptotic changes in the kidney and liver tissues that can be diminished or even terminated by the removal of the alloys from Syrian hamsters [6]. The International Agency for Research on Cancer (IARC) has listed cobalt compounds as possibly carcinogenic agents to humans (Group 2B) [7]. Thus, studies on the potential adverse effects and the related health risks of cobalt in biological systems and the environment are pertinent. A recent extensive review by Leyssens et al. [8] covered the historical and contemporary cobalt sources in different exposure settings and the related intake routes, kinetics, underlying toxicity mechanisms, and a critical evaluation of the known systemic human health effects, providing a comprehensive quantitative exposure and risk assessment. The systemic health effects are likely to occur at blood Co concentrations higher than 300 μg/L [8]. Mostly, cobalt toxicity investigations have been mainly performed for its genotoxicity [9], tissue hypoxia [10,11], and cytotoxicity [12].

Nutraceuticals have been extensively studied in recent years with the goal of finding safe therapeutics of natural origin instead of relying only on pharmaceuticals. Nutraceuticals are constituted of phytocomplexes if they are derived from a plant origin, and a blend of the secondary metabolites if they are derived from an animal origin [13]. Within nutraceuticals of vegetal origin, more than 8000 phenolic constituents were characterized, representing one of the most widely distributed class of plant secondary metabolites. The main concern regarding polyphenols is related to their high potential application for food preservation and for therapeutic beneficial use [14]. Besides, the use of natural antioxidant products from plant sources has shown promising evidence in ameliorating the toxicity of diverse xenobiotics in in vivo models [15,16,17,18,19].

*Moringa oleifera* (MO) *Lam.* is grown in many countries of the tropics and sub-tropics. Almost every part of the MO tree is useful as food supplements, traditional medicine, and for industrial purposes. The wide use of MO is due to its highly desirable nutritional content, including vitamins, amino acids, minerals, and fatty acids, besides various types of antioxidants including flavonoids, ascorbic acid, glucsinolates, carotenoids, and phenolics. The leaves of MO are high in nutritional constituents because of the relatively low moisture content compared with most vegetables [20]. A wide degree of safety of different MO extracts was reported, such as for example the median lethal dose (LD_50_) of alcoholic extract of MO leaves up to 5 gm/ kg body weight (bw) [21]. in vivo human and animals as well as in vitro studies proved that various extracts of MO leaves possess different therapeutic effects, such as anti-inflammatory, hypolipidemic, antioxidant, anti-diabetic, and chemoprotective (hepatic, cardiac, renal, and nervous) activities [11,22,23,24,25].

Oxidative stress has an important role in the development and progression of the inflammatory response to various pollutant exposure; therefore, the reciprocal relationship between oxidative injury and inflammatory processes was considered [26]. Oxidants potentiate all phases of the inflammatory process, including the production of pro-inflammatory cytokines, activation of the signaling pathways, and the adaptive cellular response [27]. The present investigation was planned to assess the role of the ethanolic extract of *Moringa oleifera* leaves (MOEE) in protecting the kidney tissue against CoCl_2_ toxicity in rats. Moreover, we assessed the changes in the expression of genes that act as convenient molecular endpoints for the inflammatory response, where the generation of reactive oxygen species (ROS) is closely linked to inflammation-triggered pathways.

## 2. Material and Methods

### 2.1. Ethical Statement

The authors declared that all procedures in the present study were conducted in strict compliance with the ethical standards of the National Institutes of Health (NIH) guidelines on the care and use of laboratory animals. Prior approval was provided from the Institutional Animal Care and Use Committee of Zagazig University (ZU-IACUC/2/F/80/2018). All efforts were considered to reduce the suffering of animals prior to and throughout the experiment and sampling.

### 2.2. Tested Chemicals and Plant Extract

Cobalt dichloride (CoCl_2_ × 6H_2_O) (CAS No. 7791-13-1) with 97% purity was obtained from Sigma-Aldrich Co. (St. Louis, MO, USA). *Moringa oleifera* (MO) plant was kindly provided from the Egyptian Scientific Society of Moringa. The botanical identification of the MO plant leaves was performed by Prof. Abou El Fetooh Abd-Allah, Chairman of the Egyptian Scientific Association for Moringa, National Research Center, Cairo, Egypt.

### 2.3. Preparation of the Ethanolic Extract of the Leaves of Moringa oleifera

**Figure 1:**
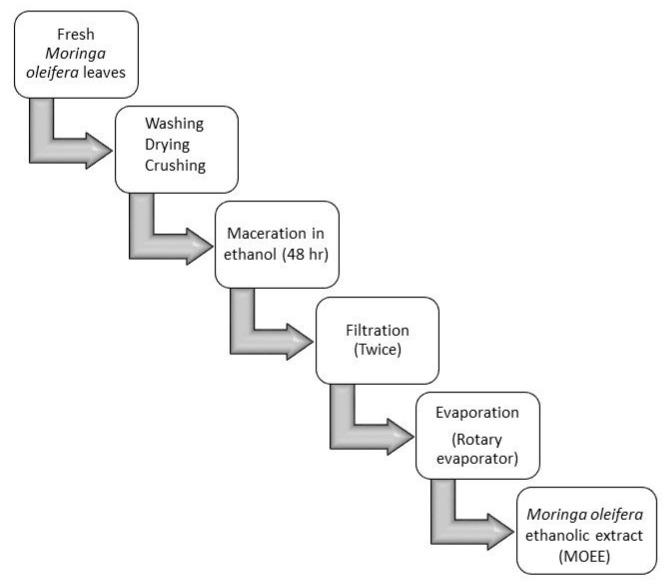
Scheme of procedure for preparation of *Moringa oleifera* ethanolic extract (MOEE).

The MOEE was prepared as follows: The plant leaves were cleaned by washing with distilled water and dried at room temperature for 21 days. The dried MO leaves were crushed into fine powder by a high-speed milling machine. Then, 1000 mg from the obtained powder was extracted in 1000 mL of ethanol (absolute) for 48 h and filtered through filter paper (2 µm-sized pores) for two times. The resultant ethanolic extract was evaporated using a rotary evaporator at 50 °C. The residual yield of the obtained MOEE extract was 78.3 gm per 1000 g of dried powder. The obtained extract was reconstituted in a brown bottle (1 gm of extract: 10 mL distilled water) and was stored at 4 °C until use (Figure 1).

### 2.4. Gas Chromatography/Mass Spectrometry Analysis (GC–MS) of MOEE Bioactive Chemical Constituents

MOEE was subjected to GC-MS analysis using the model instrument, Trace GC 1310-TSQ mass spectrometer (Thermo Scientific, Austin, TX, USA) with a direct capillary column TG–5MS (30 m × 0.25 mm × 0.25 µm film thickness). The column oven temperature was initially held at 55 °C and then increased by 5 °C/min to 200 °C with a two-min hold; then, it was increased to 300 with 20 C/min and held for 3 min. The injector temperature was kept at 270 °C. Helium (99.9%) was used as a carrier gas at a constant flow rate of 1 mL/min. The solvent delay was 2 min, and 1 µL of sample was injected automatically using an Autosampler AS3000 coupled with GC in the split mode. Electron ionization (EI) mass spectra were collected at 70 eV ionization voltages over the range of m/z 50–600 in full scan mode. The ion source and transfer line temperatures were set at 200 °C and 270 °C respectively. The elution time allowed for identifying compounds is 40 min. The components were identified by comparison of their retention times and mass spectra with those of the two important commercial libraries, the National Institute of Standards and Technology (NIST) mass spectral library (version 11) contains the EI spectra of more than 200,000 compounds (213,000 spectra), and the Wiley Registry (9th edition) contains the EI spectra of almost 600,000 unique compounds (662,780) [28].

### 2.5. Experimental Animals and Procedures

**Figure 2:**
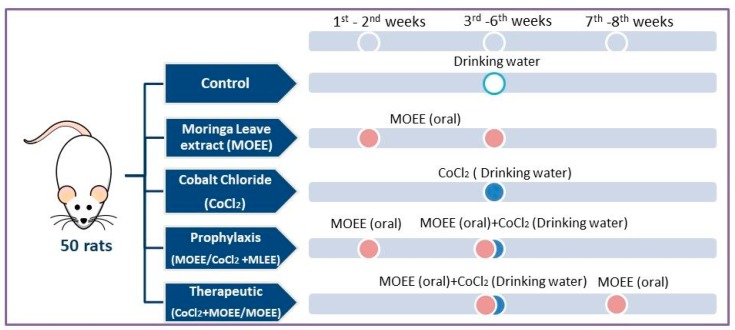
Experimental groups and treatments: MOEE: *Moringa oleifera* leave ethanolic extract (400 mg/kg body weight (bw), orally) and CoCl_2_: cobalt chloride (300 mg/L in drinking water).

The experiment was performed in 50 Sprague–Dawley male rats weighing 145–160 gm. Animals were caged under constant conditions for 10 days (21–24 °C, 50–60% relative humidity), where they had access to food and water *ad libitum*. Experimental animals were classified into five groups, where the number of animals used per each treatment was kept to a minimum of 10. The 1st group (Control) received only distilled water via oral administration. The 2nd group (MOEE) orally received 400 mg MOEE/kg bw/day for six weeks [11]. The 3rd group (CoCl_2_) received 300 mg/L freshly prepared CoCl_2_ solution in drinking water for four weeks [29]. Rats in the 4th group (prophylactic; MOEE/CoCl_2_ + MOEE) received MOEE for two weeks prior to and four weeks concurrently with CoCl_2_ exposure. Rats in the 5th> group (therapeutic; CoCl_2_ + MOEE/MOEE) co-received both MOEE for four weeks concurrently with and two weeks following CoCl_2_ exposure at the previously described doses and regimens (Figure 2). The selected dose of CoCl_2_ in this study was carried out based on the results of previous work of Awoyemi et al. [29], where it induced tissue damage via oxidative stress, inflammation, and apoptosis in a dose-dependent manner. The ethanolic extract of MO leaves was found to increase the protective activity of the antioxidant system and slow the pathological development of tissue alterations, which was induced by xenobiotic exposure when administrated at the level of 400 mg/kg bw [11,25].

At the end of the experimental period, the final body weight of rats in all groups was measured to estimate the changes in body weight. In addition, whole blood samples were withdrawn from the orbital plexus into collection tubes by puncturing the plexus by a capillary tube. The obtained blood was subjected to centrifugation at 3000 rpm for 15 min for serum separation; serum was aspirated and kept at −20 °C for biochemical assessment of renal tissue injury markers, protein profile, and 8-hydroxy-2-deoxyguanosine (8-OhdG). Animals of all the experimental groups were euthanized by cervical dislocation, and the renal tissue samples were dissected and weighed for calculation of relative weight. The dissected specimens were assigned into two sets; one set was used for homogenate preparation where the tissue was homogenized in phosphate buffer saline (1XPBS) (100 mg tissue/mL PBS), then centrifuged for 5 minutes (5000 × g rpm at 4 °C), and the supernatants were collected to measure tissue concentrations of the inflammatory, oxidative, and antioxidant markers. As well, the second set was immersed in liquid nitrogen and preserved at −80 °C until RNA extraction for gene expression analysis.

### 2.6. Biochemical Estimation

#### 2.6.1. Renal Injury Markers and Protein Profile

The renal injury markers (Creatinine, and urea) and protein profile (Total protein and albumin) were quantitatively estimated in the serum samples by colorimetric BIOMED Diagnostic Egy Chem kits (Badr city, Egypt), following the manufacturer’s instructions. The globulin level and albumin/globulin ratio were calculated.

#### 2.6.2. Oxidative Stress/Antioxidant Status Markers

A hydrogen peroxide (H_2_O_2_) colorimetric assay kit was used to estimate the level of H_2_O_2_ in the serum (Catalog No. MBS841818; MyBioSource, San Diego, United States). A quantitative determination of serum 8-hydroxy-2-deoxyguanosine (8-OHdG) was done by rat enzyme-linked immunosorbent assay (ELISA) (Catalog. No. KA0444; Abnova Co., Taiwan), following the producer’s guidelines. The lipid peroxidation product (Malondialdehyde; MDA) in renal tissue were estimated calorimetrically assayed as reported by Ohkawa et al. [30]. The determination of antioxidants status including catalase (CAT), superoxide dismutase (SOD) activity, and reduced glutathione (GSH) level was carried out following the previous described methods [31,32,33].

#### 2.6.3. Inflammatory Response Markers

Rat myeloperoxidase (MPO), tumor necrosis factor-α (TNF-α), and C-reactive protein (CRP) ELISA kits and nitric oxide (NO) colorimetric kits were used and supplied from MyBioSource, San Diego, USA. (Catalog No. MBS704859, MBS267737, MBS2508830, and MBS243214, respectively). The quantitative estimation of these biomarkers was done following the manufacturer’s recommended procedures.

### 2.7. Real-Time Quantitative PCR (RT-qPCR) Analysis of Genes Encoding Inflammation (Pro-Inflammatory Cytokines)

Total RNA was extracted from the frozen kidney specimens using the RNeasy Mini Kit (Qiagen, Germany) followed by the creation of the first-strand cDNA, which was reverse-transcribed from the extracted total RNA using a QuantiTect Reverse Transcription kit (Qiagen, Heidelberg, Germany). The primer sequences needed for the RT-PCR analysis are nuclear factor-kappa B (NF-kB) primers, 5-GCTTTGCAAACCTGG GAATA-3 and R: 5-CAAGGTCAGAAT GCACCAGA-3 [34]; interleukin (IL-6) primers, F: 5′- AAAGCCAGAGTC ATTCAGAGC-3′, R: 5′- GAGCATTGGAAGTTGGGGTA-3′ (NM_012589.2); and glyceraldehyde-3-phosphate dehydrogenase (GAPDH) primers, F: 5′-TGACGTGGACATCCGCAAAG-3′, R: 5′-CTGGAAGGTGGACAGCGGAGG-3′ [35].

A Rotor-Gene Q cycler (Qiagen, Germany) was used to perform the RT-qPCR analysis using QuantiTect SYBR Green PCR kits (Qiagen, Germany) and both primers of each gene. The PCR mixture components were 12.5 µL 2× SYBR Green PCR Master Mix, 1 µL of each primer (10 pmol/mL), 2 µL cDNA, and 8.5 µL RNase-free water in a total volume of 25 µL. The thermal cycling operations were adjusted as follows: NF-kB, GAPDH (40 cycles of 95 °C for 10 s, (GAPDH: 54 °C, NF-kB: 60 °C) for 10 s and at 72 °C for 15 s. IL-6 (40 cycles at 95 °C for 20 s, at 60 °C for 15 s, and at 72 °C for 15 s). The relative fold change in the expression of target genes were estimated by the comparative 2^−ΔΔCt^ method (Ct: cycle threshold) [36] with the GAPDH gene as an internal control to normalize the target gene expression level.

### 2.8. Data Analysis

The data were tested for normality using the Shapiro–Wilk W test and homogeneity of variances. One-way ANOVA test was conducted to test the statistical differences between treatment groups, using the SPSS 16.0 computer program (IBM Inc, Armonk, NY). The Tukey’s multiple comparisons post-hoc test was done later to compare the mean values among these groups. Cases of significance were assumed on a *p*-value of less than 0.05.

## 3. Results

### 3.1. GC-MS Profiling of the MOEE

**Figure 3:**
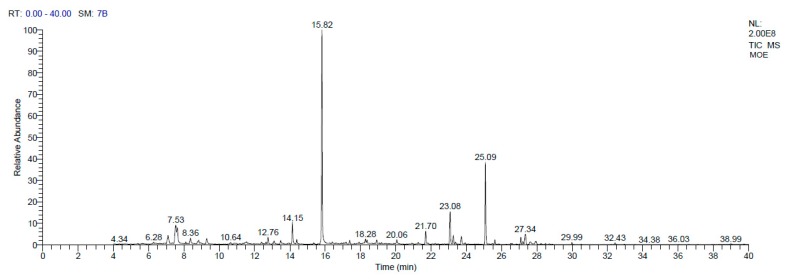
GC-MS based chemical profiling of MOEE.

The various bioactive compounds of the extract were characterized and identified by GC-MS analysis. According to GC-MS results, MOEE consists of 27 chemical components with different retention times, belonging to different chemical classes. The most identified components included eugenol (39.38%), α-bisabolol (15%), bisabolol oxide B (6.19%), thymol (4.74%), and menthol (3.41%). The other components included d-carvone (1.36%), malonic acid (1.28%), neophytadiene (1.23%), and cinnamaldehyde (1.04%) (Figure 3, Table 1).

### 3.2. Mortalities, Signs, Body Weight Changes, And Relative Kidney Weight

There were no recorded mortalities among all experimental rats. The CoCl_2−_exposed rats showed signs of dullness, fatigue, and lethargy. The degree of severity of the noticed signs was attenuated in both groups that were co-administered with MOEE (prophylactic and therapeutic). Exposure to CoCl_2_ was associated with a significant decrease in the body weight gain but no difference in the relative kidney weight in intoxicated rats in comparison with control rats. The inclusion of MOEE increased the body weight gain in the MOEE-supplemented group. Co-treatment of CoCl_2_-exposed rats with MOEE significantly improved the body weight gain in the prophylactic co-treated group compared with the CoCl_2-_intoxicated group.

### 3.3. Renal Injury Markers and Protein Profile

CoCl_2_ exposure was associated with a significant increase in the levels of urea and creatinine, compared to the control group. The co-administration of MOEE with CoCl_2_ significantly reduced the serum concentrations of both markers; the effects were more pronounced in the prophylactic (MOEE/CoCl_2_ + MOEE) than the therapeutic group (CoCl_2_ + MOEE/MOEE). Although the serum levels of these biomarkers in both co-treated groups were modulated, they did not normalize to the control level. Moreover, CoCl_2_ exposure was associated with significantly lower levels of total proteins and albumin compared to the control group. This decrease was improved upon the administration of MOEE in a protective regimen, while it showed no change in the therapeutic co-treated group, compared with CoCl_2_-exposed group. On the other hand, the serum levels of globulins were decreased on CoCl_2_ exposure, compared with control group. The declined globulin level was significantly modulated to be normalized in the prophylactic (MOEE/CoCl_2_ + MOEE) group, but non-significant changes were recorded in the therapeutic one compared with CoCl_2_-exposed group. The A/G ratio showed non-significant differences among all groups (Table 2).

### 3.4. Oxidative Stress/Antioxidant Status Markers

Significant elevations were found in renal tissue H_2_O_2_, malondialdehyde (MDA), and 8-OHdG concentrations in CoCl_2_-exposed rats, compared with control rats. The levels of H_2_O_2_ and 8-OhdG were significantly modulated by MOEE supplementation in both co-treated groups. This modulation was more prominent in the prophylactic group (MOEE/CoCl_2_ + MOEE); however, they still revealed a significant elevation compared with the control group. A significant modulation of the MDA level was recorded only in the prophylactic group, but not in therapeutic co-administered group in comparison with CoCl_2_-exposed rats.

The previously mentioned increases were associated with significant decreases in the renal tissue antioxidant indices (SOD, CAT activities, and GSH content) in rats exposed to CoCl_2_ compared with the control group. In addition, the administration of MOEE was found to significantly improve the reduced levels when co-treated with CoCl_2_; this effect was more pronounced in the prophylactic co-treated group than the therapeutic co-treated one (Table 3).

### 3.5. Inflammatory Response Markers in Kidney Tissue

**Figure 4:**
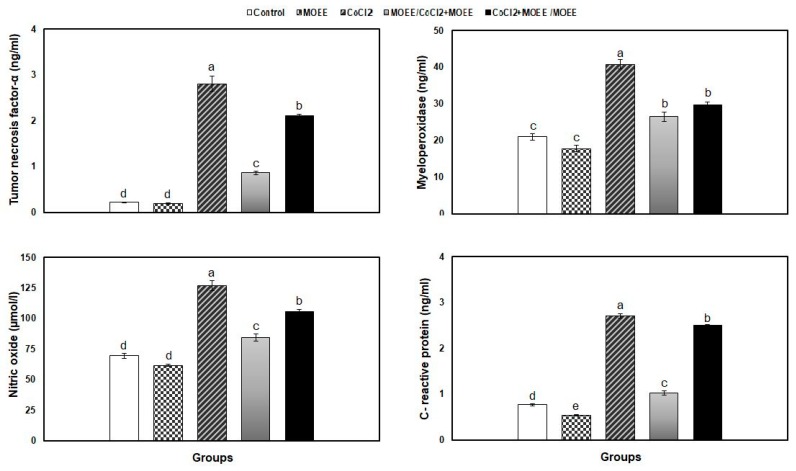
Inflammatory markers (nitric oxide, NO; tumor necrosis factor-α, TNF-α; myeloperoxidase, MPO; and C-reactive protein, CRP) in the kidney tissue of rats in response to MOEE administration (400 mg/kg bw, orally) and/or CoCl_2_ treatment (300 mg/L in drinking water). Bars carrying different letters (a, b, c, d, e) are significantly differently (*p* ˂ 0.05) (mean ± SE). One—way ANOVA followed by Tukey B *post hoc* test. MOEE: *Moringa oleifera* leave ethanolic extract-administered group, CoCl_2_: Cobalt chloride-exposed group, MOEE/ CoCl_2_ + MOEE: prophylaxis co-treated group, CoCl_2_ + MOEE/MOEE: therapeutic co-treated group.

The renal tissue concentrations of CRP, MPO, TNF-α, and NO were significantly elevated in CoCl_2_-exposed rats compared with the control rats. The administration of MOEE alone was associated with non-significant decreases in their levels, except for CRP levels, which showed a significant decrease. The co-treatment of MOEE with CoCl_2_ significantly attenuated the elevation of these indices, particularly in the prophylactic co-treated rat’s kidney more than therapeutic regimen, compared with CoCl_2_-exposed rats. Unfortunately, the recorded modulation in both co-administered groups did not attain the inflammatory response to the control value (Figure 4).

### 3.6. Relative mRNA Levels of Inflammatory Cytokines in the Kidney Tissue

**Figure 5:**
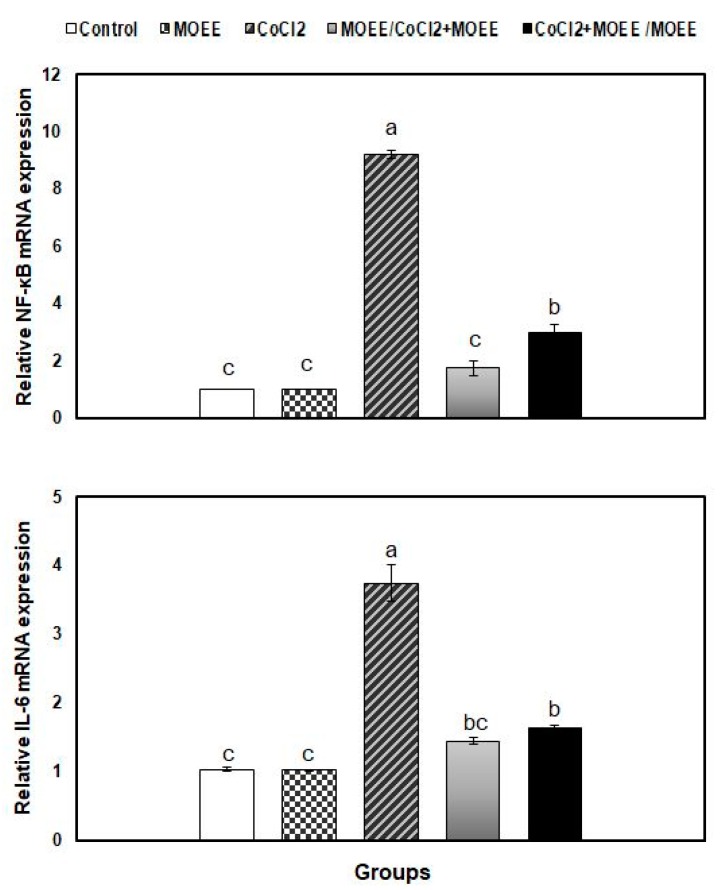
Relative expression of nuclear factor-kappa B (NF-kB) and interleukin (IL)-6 mRNA in the kidney tissue of rats in response to MOEE administration (400 mg/kg bw, orally) and/or CoCl_2_ treatment (300 mg/L in drinking water). The expression abundance of genes mRNA was normalized against the glyceraldehyde-3-phosphate dehydrogenase (GAPDH) internal control gene. Bars carrying different letters (a, b, c) are significantly different (*p* ˂ 0.05) (mean ± SE). One-way ANOVA followed by Tukey B *post hoc* test. MOEE: *Moringa oleifera* leave ethanolic extract-administered group, CoCl_2_: Cobalt chloride-exposed group, MOEE/CoCl_2_ + MOEE: prophylaxis co-treated group, CoCl_2_ + MOEE/MOEE: therapeutic co-treated group.

The relative expression of NF-kB and IL-6 in the kidney tissue was found to be significantly up-regulated in CoCl_2_-exposed rats, compared to the control group. MOEE administration significantly modulated the expression of NF-kB and IL-6 in both prophylaxis and therapeutic co-treated groups compared with the CoCl_2_-exposed group. In the prophylaxis co-treated group, such elevations were modulated to be non-significantly different from the control group, while in the therapeutic group, the down-regulated expression of the aforementioned genes was still significantly different and higher than the control group (Figure 5).

## 4. Discussion

The current study revealed the nephrotoxic effects of CoCl_2_ exposure_,_ including inflammation and oxidative stress-mediated damage. On the other hand, MOEE supplementation was associated with significant decreases in the levels of renal injury, oxidative stress, and inflammatory response indices. This modulation was associated with down-regulation of the mRNA expression profile of genes that mediate the inflammatory response in the kidney tissue. Previously, various antioxidants of natural sources have shown an ameliorative efficacy against xenobiotics-induced toxicity in in vivo animal studies [11,37,38,39,40].

The reduced body weight gains upon CoCl_2_ exposure may be explained by the metabolic cost theory where the toxic stressors induce some metabolic changes that consume the organism’s energy reserves to combat the effects of the toxicant or to activate repair mechanisms, resulting in an alteration of protein and carbohydrate metabolism [41]. On the other hand, the recorded increases in body weight gain in response to MOEE administration could be due to the desirable nutritional contents, including vitamins, amino acids, minerals, and fatty acids, besides various types of antioxidants including flavonoids, ascorbic acid, glucsinolates, carotenoids, and phenolics [25]. The phytochemical analysis of the MOEE used in the present study showed that the extract is rich in thymol that enhances the digestibility and antimicrobial efficiency against gut pathogens [42] and may be responsible for the advantageous influence of MO supplementation on animal performance. Moreover, the restoration of body weight noted in the prophylaxis co-treated group may be attributed to the growth-promoting potency of MO or the ability to hinder the oxidative injury induced by cobalt, reducing the stressful consequences of it in the exposed rats.

The nephrotoxic effects of CoCl_2_ were evidenced by the increase of serum creatinine and urea concentrations. The renal dysfunction includes the failure of the kidneys to scavenge the muscular metabolic waste product from the blood, resulting in the rise of creatinine levels in serum. In addition, when the rate of serum urea formation exceeds the rate of its clearance, the accumulation of serum urea occurs [35]. Further, alteration in the glomerular filtration, which refrains proteins and cells from being released into the urine, leads to hypoproteinemia (as recorded in the present study). Another explanation for protein content depletion is the interceding protein catabolism as an energy reserve to counter the existence stress and activate repair mechanisms [25].

Herein, MOEE co-administration with CoCl_2_ showed a nephroprotective efficiency through the improvement of the kidney function and protein synthesis. Such protection was also demonstrated against other xenobiotics, such as cadmium in rats, gentamicin in rabbits, and acetaminophen in mice [43,44,45]. Cobalt was found to accumulate in various body organs, including the liver, heart, and kidneys, in which the content of Co^2+^ correlated with the duration of exposure [46]. Therefore, the metal chelating potency against various metals such as lead, arsenic, and cobalt may explain MO protective properties against their toxic impact. This efficacy may be due to MO high calcium and ascorbic acid contents; calcium acts as an antagonist to metals/metalloids [11,47,48,49].

The deleterious influences of CoCl_2_ on the kidney tissue could be explained through the promotion of oxidative injury. Co (II) alters the cellular redox chemistry where it reacts in a Fenton-type reaction, mediating the excess production of ROS [50]. Indeed, ROS generation is a central mechanism of the genotoxicity caused by cobalt [51]. The current study showed that CoCl_2_ decreases the endogenous antioxidants’ activities and increases H_2_O_2_, MDA, and 8-OHdG formation in the renal tissue of intoxicated rats. Cobalt reacts with H_2_O_2_ in a phosphate buffer system, particularly in the existence of glutathione or histidine, to generate both superoxide and hydroxyl radicals, which damage DNA nucleotides [52]. Additionally, ROS can damage the cell membrane by reacting with the phospholipid moieties of the membrane polyunsaturated fatty acids, mediating lipid peroxidation. This was in accordance with the previous studies that demonstrated the CoCl_2_-induced oxidative injury in different body systems [11,53].

The endogenous antioxidant capacity may be altered by excessive ROS generation, causing glutathione depletion and SOD inactivation (by H_2_O_2_ and superoxide radicals). Cobalt has a mimetic action and therefore displaces divalent cations such as Zn(II) or Ca(II) that are present in the active center of the antioxidant enzymes, leading to structural and functional impairments of enzymes and signal transduction pathways [54]. The genotoxic impacts of cobalt were previously described in human and rodent cells, including chromosome aberrations, single and double-strand breaks, sister chromatid exchanges, and micronuclei [55,56]. Cobalt ions can mediate an excessive generation of ROS that induce DNA double-strand breaks directly by breaking the DNA backbone [57]. Further, owing to the high affinity of cobalt ions to sulfhydryl groups, they may inhibit the enzymes that are involved in the polymerization and incision steps of the DNA repair machinery [56].

The ability of MOEE to alleviate oxidative stress could be due to its leaves’ rich content of natural antioxidants, particularly flavonoids, including, quercetin, kaempferol, apigenin, and rutin [58]. Therefore, it possesses an anti-nephrotoxic potential as recorded in the case of acetaminophen-induced nephropathy [45]. Sreelatha and Padma [59] stated that MO leaf extract mitigated DNA damage in KB cells, increased their antioxidant enzymes, and ameliorated lipid peroxidation. Our GC-MS analysis showed that MOEE has several bioactive components such as thymol, bisabolol, eugenol, and cinnamaldehyde that exhibit strong antioxidant potency. The polyphenol eugenol was the main component in the tested extract. It possesses potent antioxidant activities owing to its phenolic group. Measuring the antioxidant activity of eugenol using 2,2’-azino-bis-3-ethylbenzothiazoline-6-sulfonic acid (ABTS) and 2,2-diphenyl-1-(2,4,6-trinitrophenyl) hydrazyl (DPPH) assays revealed that eugenol eliminates 76.9% and 90.8% of ABTS and DPPH free radicals, respectively [60]. The multiple free-radical scavenging testing method for five ROS (hydroxyl radical, superoxide, alkoxyl radical, peroxyl radical, and singlet oxygen) revealed a high singlet oxygen scavenging activity for eugenol [61]. Eugenol suppresses the production of superoxide anion by neutrophils via the inhibition of the Raf/MEK/ERK1/2/p47phox-phosphorylation pathway [62]. Moreover, it protects against ototoxicity and neurotoxicity induced by cisplatin and aluminium, respectively, via enhancing the activities of antioxidant enzymes and lowering the lipid peroxidation and 8-OHdG levels [63,64]. In vitro, the pretreatment of HCT116 cells with eugenol, 2 h prior to the mycotoxin citrinin exposure, significantly inhibited ROS generation, modulated the antioxidants enzymes activities, and reduced MDA production, protein-bound sulfhydryls level, and DNA fragmentation [65].

Another component, thymol, has well-described antioxidant, anti-inflammatory, anti-apoptotic, and organ-protective activities. It exhibited strong antioxidant properties against ketoprofen-induced pancreatic damage and inhibited DNA damage through the reduction of ROS and 8-OHdG levels [66]. (−)-α-Bisabolol has been found to possess a high reducing activity that is directly proportional to its antioxidant potential [67]. It showed the ability to lessen oxidative stress and delay the development of various disease complications by countering ROS production as in isoproterenol-induced myocardial infarction [68]. Moreover, (−)- α-bisabolol has exhibited in vivo and in vitronephroprotective activity in kidney ischemic–reperfusion models in vivo, through the attenuation of functional alterations and reduction of lipid peroxidation and urinary kidney injury molecule-1 (uKIM-1) levels, which was associated with an improvement of GSH levels in male Wistar rats, and in vitrothrough enhancing cell viability and decreasing ROS production in Rhesus Monkey Kidney Epithelial Cells (LLC-MK2 cells) [69] and inhibiting NADPH oxidase enzyme activity in HK2 cells [70]. In addition, cinnamaldehyde inhibits ROS production in association with the up-regulation of the antioxidative enzyme heme-oxygenase 1 (HO-1). Further, it reduced the accumulation of MDA in vivo and accelerated the repair of UVB-mediated DNA damage in human keratinocytes in vitro[71]. Other authors showed that cinnamaldehyde ameliorated the high glucose-induced oxidative stress and apoptosis in cultured cardiomyocytes in a Transient receptor potential ankyrin subtype 1/ nuclear factor erythroid 2-related factor (TRPA1/Nrf2) pathway-dependent manner and mitigated cardiac oxidative stress and fibrosis in mice via an increased expression of antioxidant enzymes (HO-1, glutathione peroxidase-1 (GPx-1), quinone oxidoreductase-1 (NQO-1), and catalase) [72].

From the present findings, CoCl_2_ exposure leads to an increment of H_2_O_2_, reflecting the excess ROS formation, which facilitates the inflammatory response through improving the NF-κβ- signaling pathway in rat’s kidney. This was evidenced by the up-regulation of the mRNA expression pattern of the pro-inflammatory cytokine (IL-6) and NF-κβ encoding genes, an improvement of NO production, and an elevation of MPO, TNF-α, and CRP contents in the kidney. CoCl_2_ intoxication was associated with an oxidative damage-induced renal injury, which triggers secondary inflammatory events accompanied with cytokine release, tubular infiltration of leukocytes, and further exaggerating the renal injury [73]. Oxidative stress may actuate both cellular death and extracellular matrix breakdown (ECM). Consequently, necrotic cells and damaged ECM release different intracellular and extracellular molecules that stimulate inflammatory cascades via recognition by pattern recognition receptors (PRRs) [74]. Additionally, oxidative stress may actuate impairments within lipids and proteins, forming oxidation-specific epitopes that act as potential damage-associated molecular patterns ready to mediate innate immune cell responses to pro-inflammatory stimuli through binding to various PRRs.

NF-κB has a strategic position at the crossroad between oxidative stress and inflammation; it was proposed that ROS might represent key secondary mediators responsible for the activation of NF-κB in response to multiple stimuli [75]. IL-6 is an important mediator of the inflammatory response as it participates in the development and differentiation of B- and T-cells, as well as the activation of acute phase proteins [76]; this was supported by the high level of CRP in the present study. Previous in vitro studies revealed that CoCl_2_ induces a pro-inflammatory response by increasing the expression of several pro-inflammatory chemokines as TNF-α and IL-6, which usually occur in response to NF-kB activation [77].

The elevated content of NO− induced cytotoxicity by reacting with other free radicals yielding the highly reactive peroxynitrite radical, which oxidizes cellular biomolecules such as DNA, lipids, and protein [78]. Additionally, the generation of large amounts of NO in the glomerulus can enhance the progression of renal failure in several forms of glomerulonephritis [79]. Moreover, the obtained results revealed that CoCl_2_ exposure significantly induced MPO activity (a marker for neutrophil infiltration), as when neutrophils are activated, they release MPO into the extracellular space during the inflammatory process. MPO catalyzes the synthesis of hypochlorous acid, which is a toxic agent to cellular components, intensifying the oxidative injury [80]. Histopathologically, Akinrinde et al. [81] observed an inflammatory cell infiltration in the renal tissue of rats exposed to cobalt.

The results of the present study showed that MOEE alleviated CoCl_2_-induced inflammation in co-treated rats and protected against the inflammatory-mediated exacerbation of renal damage in CoCl_2_ intoxicated rats. This suggests that MOEE combats the production of pro-inflammatory cytokines by suppressing the NF-κB pathway through the down-regulation of IL-6 and NF-κB expression. Similarly, MOEE pretreatment of bovine mammary epithelial cells stimulated with lipopolysaccharide (LPS) blocked the activation of NF-κB [82]. Minaiyan et al. found that MO prevented inflammatory cells’ infiltration and blocked the release of pro-inflammatory cytokines through the suppression of the NF-κB signaling pathway in experimental colitis [83].

Herein, the anti-inflammatory activities of MOEE are possibly attributed to its constituents of bisabolol, thymol, caryophyllene, and eugenol. Fontinele et al. [84] and Cavalcante et al. [85] demonstrated that treatment with (-)-α-bisabolol reduced the inflammation in inflammatory pain and systemic infection experimental models in mice by the modulation of the release of pro- and anti-inflammatory cytokines and reducing NO production. It significantly prevented the increase of MPO activity, TNF-α, and inducible nitric oxide synthase (iNOS) production in permanent focal cerebral ischemia [86]. Moreover, it attenuates inflammation by inhibiting nucleotide-binding oligomerization-like receptors P3 (NLRP3) inflammasome activation and Toll Like Receptor 4 (TLR4)-NFκB/ mitogen-activated protein kinases (MAPK) signaling pathways [87]. Eugenol is able to resist the release of pro-inflammatory mediators in lipopolysaccharide-induced injury [88,89]. β-Caryophyllene, a bicyclic sesquiterpene, has been reported to exert an anti-inflammatory activity, as it inhibited mRNA expression of iNOS, IL-1β, IL-6, and Cyclooxygenase-2 (COX-2) in C6 microglial cells [90]. Moreover, previous studies have stated that thymol can elicit an anti-inflammatory action by suppressing pro-inflammatory signaling pathways and transcription factors such as p38 mitogen-activated protein kinases (MAPK), signal transducer and activator of transcription3 (STAT-3), NF-κB and activator protein-1 (AP-1), and TLR4-mediated Ras homolog family member A (RhoA)-dependent NF-κB in LPS-induced inflammation [91,92,93].

## 5. Conclusions

The current study showed that CoCl_2_ exposure induced oxidative stress, DNA damage, and inflammation in the kidney tissue of rats, indicating a nephrotoxic impact. However, MOEE administration (particularly in a prophylactic regimen) inhibited CoCl_2_-induced nephrotoxicity, probably via mitigating DNA damage, ROS mediated-oxidative injury, and inflammation (through modulating the expression pattern of targeted genes. Therefore, MOEE could be a good candidate for a supplement to alleviate oxidative injury and inflammatory responses, which occur in renal toxicities and pathological disorders. Nevertheless, further studies are needed to elucidate the molecular mechanisms underlying MOEE and its constituents’ potency against the toxicities elicited by cobalt exposure.

## Figures and Tables

**Table 1:** Bioactive chemical constituents assigned in MOEE by GC-MS analysis.

Bioactive Chemical Constituents	Chemical Formula	Mass Weight (MW)	Matching Factor (MF)	Retention Time (RT) (min)	Area %
Eugenol: 4-allyl-2-methoxyphenol	C_10_H_12_O_2_	164	951	15.82	39.38
α-Bisabolol	C_15_H_26_O	222	877	25.09	15.00
Bisabolol oxide B: 2-Furanmethanol,tetrahydro-à,à,5-trimethyl-5-(4-methyl-3-cyclohexen-1-yl)-, [2S-[2à,5á(R*)]]-	C_15_H_26_O_2_	238	927	23.08	6.19
E-dipropylene glycol	C_6_H_14_O_3_	134	943	7.53	4.84
Thymol: 2-isopropyl-5-methylphenol	C_10_H_14_O	150	918	14.15	4.74
Menthol: 1’-(butyn-3-one-1-yl)-, (1S,2S,5R)- 4-(1-Hydroxy-2-isopropyl-5-methylcyclohexyl)-3-butyn-2 -one	C_15_H_24_	204	701	21.70	3.41
2-(2-Hydroxypropoxy)-1-propanol	C_6_H_14_O_3_	134	950	7.62	3.30
7-Acetyl-6-ethyl-1,1,4,4-tetramethyltetralin	C_18_H_26_O	258	930	27.34	2.78
2-Propanol,1,1-oxybis-	C_18_H_32_O_2_	134	892	7.10	2.67
(-)-α-Bisabolol oxide A: 2H-Pyran-3-ol, tetrahydro-2,2,6-trimethyl-6-(4-methyl-3-cyclohexen-1-yl)-, [3S[3alpha,6alpha (R*)]]	C_15_H_26_O_2_	236	845	23.72	1.80
7a-Isopropenyl-4,5-dimethyloctahydroinden-4-yl)methanol	C_15_H_26_O	222	816	23.26	1.53
2-phenylethanol	C_8_H_10_O	122	921	9.29	1.39
D- Carvone: 2-Cyclohexen-1-one, 2-methyl-5-(1-methylethenyl)-	C_10_H_14_O	150	938	12.76	1.36
Malonic acid: bis(1-methyl-2-hydroxyethyl)ether	C_3_H_4_O_4_	104	999	8.36	1.28
Neophytadiene: 1,3-butadiene, 2-(4,8,12-trimethyltridecyl)-	C_20_H_38_	278	917	27.09	1.23
(E)-Cinnamaldehyde: trans-3-phenyl-2-propenal	C_9_H_8_O	132	871	13.47	1.04
1H-3a,7-Methanoazulene,2,3,6,7,8,8a-hexahydro-1,4,9,9-tetramethyl-(1à,3aà,7à,8aá)-	C_15_H_24_	204	868	18.28	0.97
Phthalic acid: 1,2-Benzenedicarboxylic acid, diethyl ester	C_12_H_14_O_4_	222	942	20.06	0.96
1-Chloro-7-heptadecyne	C_17_H_31_Cl	270	743	27.96	0.90
1-chlorotetradecane	C_14_H_29_Cl	232	733	8.83	0.87
Acetyl cedrene, Ethanone	C_17_H_26_O	246	940	25.62	0.82
Nerol: (Z)-3,7-dimethyl-2,6-octadien-1-ol	C_10_H_18_O	154	907	13.11	0.80
Methyl pimar-8-en-18-oate	C_21_H_34_O_2_	318	623	27.62	0.76
Caryophyllene: Bicyclo[7.2.0]undec-4-ene, 4,11,11-trimethyl-8-methylene-, [1R-(1R*,4E,9S*)]-	C_15_H_24_	204	888	17.39	0.68
Alloaromadendrene: (1aS,4aR,7S,7aS,7bR)-1,1,7-Trimethyl-4-methylendecahydro-1H-cyclopropa[e]azulen	C_15_H_24_	204	907	18.37	0.65
α-Isomethyl ionone: 4-(2,6,6-Trimethyl 2-cyclohexen-1-yl)-3-methyl-3-buten-2-one	C_14_H_22_O	206	890	18.92	0.64
2-Pentadecanone, 6,10,14-trimethyl-	C_18_H_36_O	268	876	27.21	0.43

**Table 2:** Body weight change, relative kidney weight, kidney tissue injury markers, total protein variables in rats in response to MOEE administration (400 mg/kg body weight (bw), orally) and/or CoCl_2_ treatment (300 mg/L in drinking water).

Parameters	Experimental Groups				
	Control	MOEE	CoCl_2_	MOEE/CoCl_2_+MOEE	CoCl_2_+MOEE /MOEE
Body weight change (gm)	12.00 ^b^ ± 1.09	18.16 ^a^ ± 1.44	−11.00 ^d^ ± 0.73	−5.00 ^c^ ± 2.67	−8.00 ^cd^ ± 0.57
Relative kidney weight	0.25 ± 0.01	0.26 ± 0.01	0.27 ± 0.02	0.26 ± 0.02	0.28 ± 0.02
Kidney tissue injury markers					
Urea (mg/dL)	21.18 ^d^ ± 0.80	20.54 ^d^ ± 0.61	43.48 ^a^ ± 1.69	26.65 ^c^ ± 1.15	37.51 ^b^ ± 1.56
Creatinine (mg/dL)	0.83 ^d^ ± 0.02	0.84 ^d^ ± 0.04	1.97 ^a^ ± 0.06	1.09 ^c^ ± 0.05	1.49 ^b^ ± 0.06
Protein profile					
Total protein (gm/dL)	6.47 ^a^ ± 0.25	6.43 ^a^ ± 0.29	3.97 ^c^ ± 0.29	5.39 ^b^ ± 0.21	4.20 ^c^ ± 0.31
Albumin (gm/dL)	3.97 ^a^ ± 0.23	4.09 ^a^ ± 0.23	2.34 ^c^ ± 0.16	3.24 ^b^ ± 0.08	2.56 ^bc^ ± 0.22
Globulin (gm/dL)	2.49 ^a^ ± 0.09	2.35 ^a^ ± 0.13	1.63 ^b^ ± 0.15	2.15 ^ab^ ± 0.18	1.64 ^b^ ± 0.12
A/G ratio	1.60 ± 0.09	1.75 ± 0.11	1.46 ± 0.07	1.56 ± 0.14	1.56 ± 0.12

Means within the same row bearing different superscripts (a,b,c,d) are considered significantly varied (*p* ˂ 0.05) (mean ± SE). One-way ANOVA followed by Tukey’s B *post hoc* test. MOEE: *Moringa oleifera* leave ethanolic extract, CoCl_2_: Cobalt chloride-exposed group, MOEE/ CoCl_2_+MOEE: prophylaxis co-treated group, CoCl_2_ + MOEE/MOEE: therapeutic co-treated group.

**Table 3:** Antioxidants and oxidative stress variables in rats in response to MOEE administration (400 mg/kg bw, orally) and/or CoCl_2_ treatment (300 mg/L in drinking water).

Parameters	Experimental Groups				
	Control	MOEE	CoCl_2_	MOEE/CoCl_2_+MOEE	CoCl_2_ + MOEE /MOEE
**Antioxidants biomarkers**					
SOD activity (U/gm tissue)	3.77 ^a^ ± 0.31	3.82 ^a^ ± 0.23	1.63 ^c^ ± 0.19	2.48 ^b^ ± 0.12	1.81b ^c^ ± 0.20
CAT activity (U/gm tissue)	1.14 ^a^ ± 0.03	1.21 ^a^ ± 0.05	0.54 ^d^ ± 0.04	0.97 ^b^ ± 0.01	0.73 ^c^ ± 0.05
GSH level (mmol/gm tissue)	0.56 ^a^ ± 0.03	0.55 ^a^ ± 0.03	0.21 ^d^ ± 0.02	0.42 ^b^ ± 0.02	0.32 ^c^ ± 0.01
**Oxidative injury biomarkers**					
H_2_O_2_ (ng/mL)	1.12 ^d^ ± 0.02	1.06 ^d^ ± 0.03	2.77 ^a^ ± 0.03	1.66 ^c^ ± 0.04	2.22 ^b^ ± 0.05
MDA (nmoL/gm tissue)	9.97 ^c^ ± 0.12	9.49 ^c^ ± 0.38	25.06 ^a^ ± 2.23	15.18 ^b^ ± 1.12	21.04 ^a^ ± 0.70
8OHdG (ng/mL)	0.09 ^d^ ± 0.01	0.07 ^d^ ± 0.01	0.37 ^a^ ± 0.02	0.16 ^c^ ± 0.01	0.31 ^b^ ± 0.01

Means within the same row bearing different superscripts (a,b,c,d) are considered significantly varied (*p* ˂ 0.05) (mean ± SE). One-way ANOVA followed by Tukey’s B *post hoc* test. MOEE: *Moringa oleifera* leave ethanolic extract, CoCl_2_: Cobalt chloride-exposed group, MOEE/ CoCl_2_ + MOEE: prophylaxis co-treated group, CoCl_2_ + MOEE/MOEE: Therapeutic co-treated group, 8OHdG: 8-hydroxy-2-deoxyguanosine, CAT: catalase, GSH: glutathione, SOD: superoxide dismutase.
